# Case Report: Case report on multiple extradural thoracic lesions with myelopathy as the clinical presentation in a systemic sarcoidosis- another tale of a lurking entity

**DOI:** 10.12688/f1000research.13553.1

**Published:** 2018-01-03

**Authors:** Sunil Munakomi

**Affiliations:** 1 Department of Neurosurgery, Nobel Teaching Hospital, Biratnagar, 0977, Nepal

**Keywords:** neuro-sarcoidosis, myelopathy

## Abstract

Herein, we report a rare case study of a sarcoidosis presenting with the features of compressive myelopathy. There were multiple extra-dural lesions in the thoracic region. Computerized Tomography (CT) of the chest revealed fibrotic changes with a pleural based nodular lesion in the right lung. The patient underwent laminectomy and partial excision of both the lesions. The histology revealed presence of non-caseating granulomas. The patient made a good recovery following adjuvant medical management with steroid and Methotrexate. Repeat CT scan of the chest also confirmed good resolution in the size of the pleural based nodule.

## Introduction

The involvement of the spine due to sarcoidosis is a rare occurrence ^1^. The involvement of an extradural region, and its presentation with features of compressive myelopathy, is an even more uncommon epi-phenomenon ^2^. Herein, we present a case study of a 50 year old male patient, who had collaborative radiological and histological evidence of an extradural spinal sarcoidosis with pulmonary involvement. The patient showed good improvement following therapy with steroids and Methotrexate. This highlights the clinical implication of keeping such entities in the differential diagnosis in patients presenting with myelopathy for better therapeutic benefits. Though a rarity, sarcoidosis still remains a lurking entity with higher tendency for multisystem involvement.

## Case study

A 50-year old male farmer, presented for the first visit to our spine clinic in December 2017 with a history of slowly progressing weakness of his bilateral lower limbs over last 4 months. Due to financial restrain, he decided not seeking medical help for his symptoms. However, in recent weeks, he was not even able to stand on his own, thereby restricting him from carrying out even most basic activities of his daily life. The patient declared no history of trauma or fall injuries. There was no fever, chronic cough, significant weight loss, or swellings elsewhere in the body, or any bladder or bowel symptoms. He had no relevant history of close contact with patients having Tuberculosis, which is among the major causes of myelopathy in our context. There was no relevant past medical or surgical illnesses, or family history of any malignancies or similar ailments.

**Figure 1.**
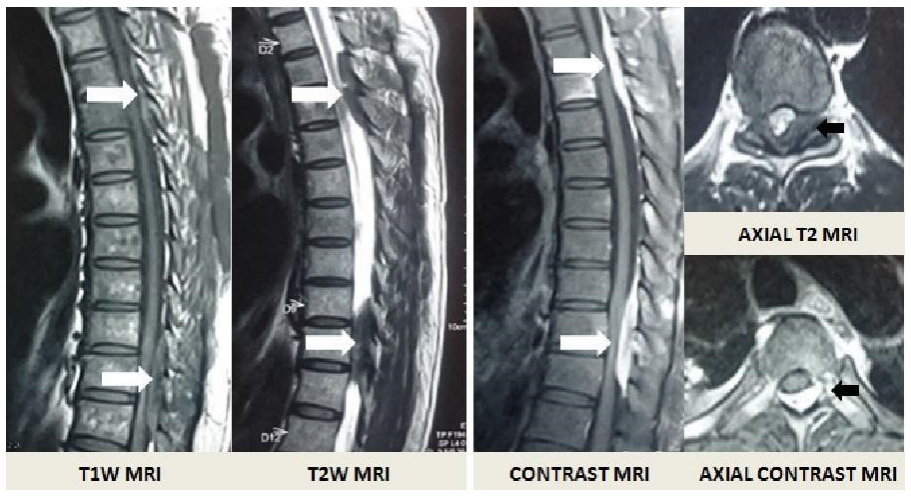
MRI images revealing the presence of multiple extradural enhancing lesions in the thoracic region.

The patient was well built, conscious and well oriented to time, place and person. There was no pallor or lymphadenopathy. His vision, as well as all cranial nerves was intact. Spinal examination revealed presence of sensory loss below T7 with exaggerated knee reflexes and up-going Babinski sign suggesting myelopathy. Vibration sense was disturbed from the D4 spinous process. Systemic examination was clinically normal. Urgent Magnetic Resonance Imaging (MRI) of the spine revealed the presence of multiple enhancing extradural lesions compressing the cord in the D3-D4 and D9-D10 thoracic region ( Figure 1). There were posteriorly based lesions with iso-hypointense in the T1 image, hypointense in the T2 image and homogenously enhancing following contrast administration.

**Figure 2.**
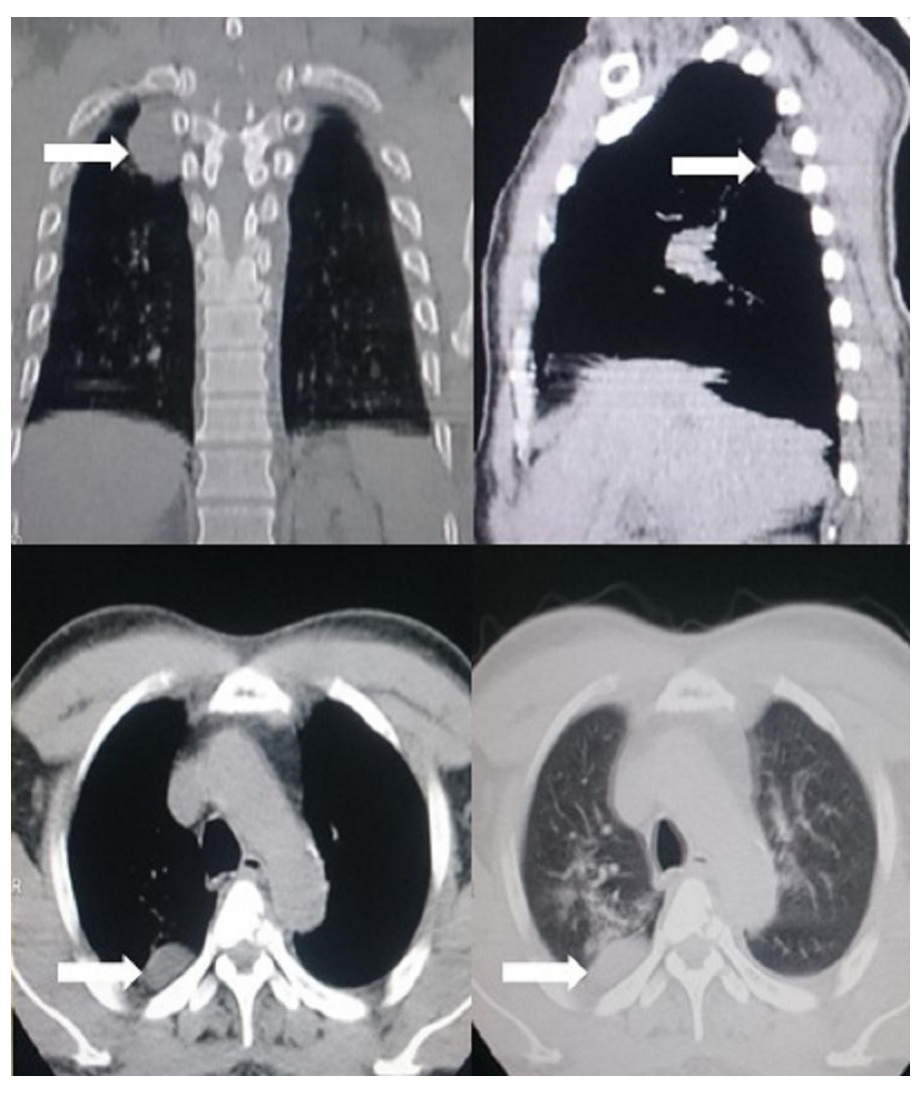
CT images revealing presence of fibrotic changes along with a pleural based nodule in the right lung.

We had an initial differential diagnosis of multiple metastatic lesions to the spine with features of compressive myelopathy. Ultrasound of the abdomen was normal. Computerized Tomography (CT) of the chest, however, revealed multiple fibrotic changes in the right upper lung fields with a posteriorly based pleural nodule ( Figure 2). Since the patient was having compressive lesions with deteriorating neurological status, we advised the patient on the urgent need of surgical decompression and a biopsy of the lesions in planning its subsequent management.

**Figure 3.**
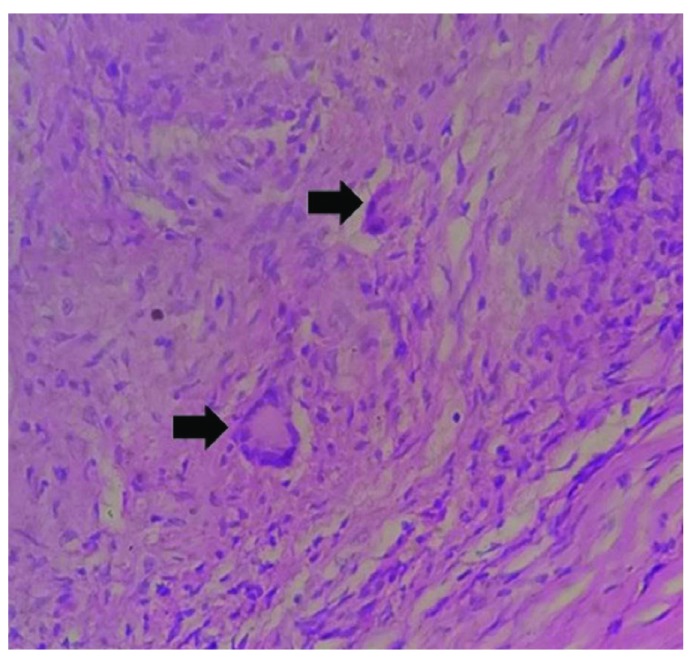
Histology revealing presence of multiple non-caseating granulomas.

After receiving written consent for the surgery, the patient underwent thoracic laminectomy. There were firm and densely adherent dural based lesions. Adequate bony decompression and subtotal resection was carried out, and then sent for histopathological study. Histology was negative for any malignant cells. It revealed presence of lymphocytes with few scattered non-caseating granulomas ( Figure 3). Acid Fast Staining (AFB) was negative. Serum Angiotensin Converting Enzyme (ACE) assays was marginally high. MRI screening of the brain was normal. We advised the patient of the significance of having a Flu-deoxy-glucose Positron Emission Tomography (FDG-PET) scan, allowing more accurate confirmation of the diagnosis. However, the financial aspect again proved to be a limiting issue. Following a thorough examination with the radio-pathological findings, the final diagnosis of a pulmonary sarcoidosis with extradural spinal involvement was made.

**Figure 4.**
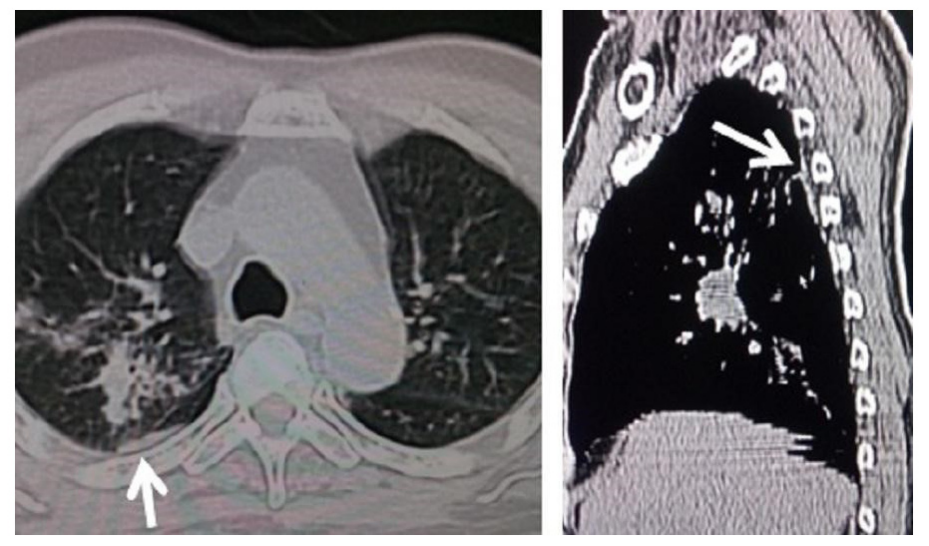
Repeat CT image revealing good resolution in the size of the pleural based nodule during the follow up at 1 month.

The patient was started on Oral Prednisolone (tapering dose starting from 40mg/day) and weekly Methotrexate (7.5mg) therapy. Since then, the patient has good neurological improvement, being able to walk independently with a walking stick within 10 days. The patient could walk independently in a follow up visit at 4 weeks. Repeat CT scans of his chest revealed good resolution on the pleural based lesion as well ( Figure 4). We advised the patient to slowly reduce the drugs within the next 4 months. He was advised about regular follow up for periodic neurological and pulmonary examinations to determine early recurrence or progression of the disease.

## Discussion

The spine is affected in only 1% of patients with sarcoidosis ^1^. As such, epidural involvement presenting as a myelopathy is even a rare entity ^2^. Cerebral spinal fluid (CSF) ACE level is not reliable enough in coming to the diagnosis in an isolated neuro-sarcoidosis without systemic involvement ^3^.

Junger *et al*. formatted imaging characteristics to diagnose spinal medullary sarcoidosis with findings of linear streaks, then the parenchymal enhancement followed by medullary changes with reduction of edema, and finally the cord atrophy ^4^. However, the most common finding seen in cases with neuro-sarcoidosis is the lepto-meningeal enhancement ^5^. FDG-PET scans may be a valuable adjunct in assessing extra-cranial lesions for biopsy in making a confirmative diagnosis ^6^. However, availability and its high costs are the major limiting factors for its regular appliance. Histological finding of a non-caseating granulomas are conclusive ^7^.

In order to facilitate the diagnosis, and thereby the subsequent management in cases with neuro-sarcoidosis, a diagnostic algorithm has been formulated ^8^.

Management with use of steroids and immunosuppressant (as a steroid sparing agent), both blocking the TNF, is the current therapeutic recommendation for systemic involvement ^9^.

Due to the paucity of such cases, difficulty in assessing their clinical behavior, and high propensity for recurrence and progression, it is prudent for a prolong follow up with clinical and radiological examinations ^10^.

Clinical ascertainment of such cases with neuro-sarcoidosis still remains a major challenge ^5^.

Our case was unique in the sense that myelopathy was the presenting feature in a spinal sarcoidosis, with pulmonary involvement as well. There were multiple extradural lesions in the thoracic region causing the compression. Urgent spinal decompression and histological study of such lesions followed by medical therapy with steroid and Methotrexate is advocated in such a case ^2^. Compressive myelopathy was the presenting feature in 19% of cases in a cohort study of 54 patients with neuro-sarcoidosis ^5^. Steroid with various immune-suppressants were used in that study, with steroid and Methotrexate utilized in 15% of the cases ^5^.

## Conclusion

This case highlights the clinical implications of keeping sarcoidosis in the differential diagnosis in patients presenting with features of myelopathy but having evidence of multisystem involvement. It is advisable for those patients to have prolonged follow ups so that an early assessment of any signs of recurrence or progression could be established.

## Consent

Written consent was obtained from the patient regarding the publication of the clinical data and the relevant radiological images.
