# Supplementary material for: Case Report: Case report on multiple extradural thoracic lesions with myelopathy as the clinical presentation in a systemic sarcoidosis- another tale of a lurking entity
Source: F1000Res. 2018 Jan 3;7:6. [Version 1] doi: 10.12688/f1000research.13553.1 (PMC6020724; doi:10.12688/f1000research.13553.1)
Supplement: Peer review history

# Peer review: Referee response for version 1

**DOI:** 10.5256/f1000research.14720.r34638

**Published:** 2018-06-28

**Authors:** Alfredo De Giorgi

**Affiliations:** 1 Department of Medical Sciences, Faculty of Medicine, Pharmacy and Prevention, University of Ferrara, Ferrara, Italy

This article reports an uncommon case report of a patient presenting with extradural thoracic lesions related to neurosarcoidosis.

 The case is appropriately presented.

 I recommend, if possible, to add a mini Literature review of previous cases for a better evaluation of the originality and rarity of the case reported.

I have read this submission. I believe that I have an appropriate level of expertise to confirm that it is of an acceptable scientific standard.

# Peer review: Referee response for version 1

**DOI:** 10.5256/f1000research.14720.r29430

**Published:** 2018-06-25

**Authors:** Sachin Anil Borkar

**Affiliations:** 1 Department of Neurosurgery, Neurosciences Centre, All India Institutes of Medical Sciences, New Delhi, Delhi, India

It is a well written manuscript regarding a rare entity. 

 It is a well-illustrated case of a rare disease presenting in a rare fashion.

 It should be kept in the differential diagnosis in TB endemic area presenting with myelopathy. 

 The manuscript can be accepted.

I have read this submission. I believe that I have an appropriate level of expertise to confirm that it is of an acceptable scientific standard.

# Peer review: Referee response for version 1

**DOI:** 10.5256/f1000research.14720.r30617

**Published:** 2018-02-22

**Authors:** Walid Radwan

**Affiliations:** 1 Department of Neurosurgery, West Virginia University, Morgantown, West Virginia, USA

The article is a case report of a patient presenting with myelopathy and found to have neurosarcoidosis. Relevant history and examination is provided. In addition, imaging findings are consistent and described accurately.

 However, with regards to recommending FDG-PET after obtaining pathological specimen it is not necessary and is not a cost-effective method in the management of this diagnosis. The diagnosis is confirmed on pathology which was obtained in this patient.

I have read this submission. I believe that I have an appropriate level of expertise to confirm that it is of an acceptable scientific standard.
